# The Influence of Match Status on Ball Possession in High Performance Women’s Football

**DOI:** 10.3389/fpsyg.2020.00487

**Published:** 2020-03-23

**Authors:** Rubén Maneiro, José L. Losada, Claudio A. Casal, Antonio Ardá

**Affiliations:** ^1^Department of Science of Physical Activity and Sport, Pontifical University of Salamanca, Salamanca, Spain; ^2^Department of Methodology of Behavioral Sciences, University of Barcelona, Barcelona, Spain; ^3^Department of Science of Physical Activity and Sport, Catholic University of Valencia “San Vicente Mártir”, Valencia, Spain; ^4^Department of Physical and Sport Education, University of A Coruña, A Coruña, Spain

**Keywords:** women’s football, performance analysis, ball possession, decision trees, match status, situational variables

## Abstract

The objective of this study was to examine the effect of the situational match status variable on the ball possession of the teams that participated in the 2015 FIFA Women’s World Cup. The 52 games played during the championship have been collected, and 3,740 ball possessions made by the teams were analyzed. The teams have been divided into successful and unsuccessful. Three types of analysis have been carried out: a univariate analysis for both groups with the categorical and continuous variables selected; a bivariate analysis, using chi-square tests and the exact Fischer test; and finally, a multivariable technique such as the decision trees was incorporated. The available results show significant differences between the two groups considered. Specifically, there are significant differences between winning and losing teams in terms of match status. The results of the *post hoc* test have shown that unsuccessful teams make few ball possessions with a winning match status, most of the possessions are performed when they are losing. Instead, successful teams make more possessions when they are winning than when they are losing. Also, spend more time keeping the ball in their offensive zone, and completing a greater number of passes in it. The results of the decision tree identified that the unsuccessful teams have more ball possessions in forward and middle lines with a draw during the first half, while in the second, a large percentage of possessions are made with an unfavorable match status. Instead, the successful teams have more ball possessions in the first part with a draw, while in the second it happens with a favorable match status.

## Introduction

The analysis of performance in men’s football is currently in an era of expansion, both quantitatively and qualitatively, as far as studies are concerned. Finding the routes or paths that help maximize the chances of a team’s success must be one of the highest aspirations of scientific research. Unlike other sports such as basketball or handball, football is a sport that is characterized by a low score on the scoreboard (Tenga et al., 2010). The study of different situational variables in football can help increase the number of goals and the success of the teams.

It has been shown that the behaviors carried out by male soccer players during matches are affected by situational variables such as match location, match status, and opposition quality (Taylor et al., 2008; Lago-Peñas, 2009; Mackenzie and Cushion, 2013; García-Rubio et al., 2015; Liu et al., 2015; Sarmento et al., 2017).

One of the situational variables analyzed in high performance football is match status (wining, drawing, or losing). The match status of a game is determined by the momentary result of the match at the time a behavior is recorded (Bloomfield et al., 2005). The effect of this situational variable in the game is manifested in the changes in the behavior of players and teams in response to the demands of the scoreboard.

These behavior changes influenced by match status affect different levels of performance. On a physical level, it has been shown that male soccer players of high performance teams perform significantly less high intensity activities when they are winning than when they are losing or drawing (Bloomfield et al., 2005; Lago et al., 2010; Castellano et al., 2011; Lago-Peñas, 2012). Some of the possible explanations of this behavior are that the players manage their physical abilities during the matches, and do not always use their maximum physical capacity during the confrontation. Lago-Peñas (2012), states that winning on the scoreboard is a comfortable state for a team, and it is possible for players to assume a ball retention strategy and slow down the game at lower speeds. On the other hand, when the result is losing, players try to achieve greater physical performance to tie or win.

At the tactical level there are also outstanding results regarding the possession of the ball. Taking the variable “ball possession” in isolation, preliminary studies have shown that the best teams maintained a higher percentage of ball possession during the matches, and that their game pattern was more stable (Lago-Peñas and Dellal, 2010). In addition, the local teams have more possession than the visiting teams (Lago and Martín, 2007), and the ball possession time is greater in the first part in the successful teams (Casal et al., 2017). In addition, the local teams have more ball possession than the visiting teams (Lago and Martín, 2007), and the ball possession time is greater in the first half of the match in successful teams (Casal et al., 2017). It has also been found that successful teams have significantly longer ball possessions than unsuccessful ones (Jones et al., 2004), and that the former maintain this ball possession in the mid-offensive zone, while the unsuccessful ones kept it in the middle defensive zone. The study of Amatria et al. (2019b), finds significant results that relate a high elaboration of possession (number of passes), with shooting actions (*p* < 0.043) and sendings to the area (*p* < 0.000). In addition, it also finds significant results that relate the ball possessions that begin in the own team’s field with goal actions (*p* < 0.023) and shot to goal (*p* < 0.000). Finally, Casal et al. (2017), collect that the probability of winning increases 1.72 times when the possession of the ball is done in the middle offensive zone.

There is much debate in scientific literature trying to relate the possession of the ball with match status. Different studies show changes in the behavior of the teams in response to the demands of the scoreboard. On the one hand, the works of Jones et al. (2004), Lago and Martín (2007), Lago-Peñas (2009), Lago-Peñas and Dellal (2010), and Barreira et al. (2011), collect that the teams that are losing on the scoreboard have longer possession periods, specifically every 11 min that the team is losing, increases its possession by 1%. Bradley et al. (2013), perform a simulation of possession, and affirm that if a team had a losing score (match status) during the 90 min of the match, the estimate of possession would be 66.97%, while if it were winning it would be 59.77%.

In contrast, the studies by Bloomfield et al. (2005), Taylor et al. (2008), and Casal et al. (2017) disagree with these results, and affirm that the longest possession time is carried out by the teams that are winning. Some of the possible causes of these differences could lie in the different playstyles of the teams and the type of competition (league championships and championships based on qualifiers).

Regarding the area of ball possession in relation to the match status, Lago-Peñas (2009) and Barreira et al. (2011) state that with a winning match status, teams have more possession in defensive zones, and a losing match status increases possession in the offensive third of the field. Almeida et al. (2014), and Santos et al. (2017) confirm that unsuccessful teams defend in more advanced areas of the field. On the other hand, the study of Casal et al. (2019), differentiate between successful and unsuccessful teams in the UEFA Euro 2016, and the duration of the ball possessions. They observe that unsuccessful teams have longer possessions when they are losing on the scoreboard, while in the successful teams the match status variable does not influence the ball possession time. In addition, at a predictive level they found that, in the case of successful teams, longer possessions in the offensive zone with the score in favor are performed and, in the defensive zone with a draw score. For teams that do not succeed, the possessions will be longer in the defensive zone with a draw score.

In view of the data, it is possible to verify that the situational match status variable modifies the behavior carried out by the male soccer teams during competitions. On the other hand, this variable has not yet been verified in women’s football. Therefore, the objective of this study was to know the behavior of the match status variable and how it modulates the possession of the ball in high level women’s football. For this, three types of analysis were carried out: first, a univariate analysis with the categorical and continuous variables selected; secondly, through chi-square tests and Fischer’s exact test, the aim was to study the relationship between the variables described, taking as reference the match status variable; finally, a multivariable technique based on decision trees was incorporated, proposing different success models.

## Materials and Methods

### Design

Among the possible designs that observational methodology can present, a nomothetic, intersessional follow-up and multidimensional design was applied (Anguera, 1979). The systematic observation carried out has been non-participant and active, using an observational sampling “all occurrence.”

### Sampling

In order to control the situational variables that could be affected by the match status between successful and unsuccessful teams, all matches corresponding to the FIFA Women’s World Cup 2015 have been analyzed. In this study, the unit of analysis is ball possession in high level football. Encounters were recorded from public images broadcasted on television, and through a post-event record, thus ensuring respect for behavior spontaneity, as well as the registration in its natural environment. According to the National Commission for the Proptection of Human Subjects of Biomedicaland Behavioral Research (1978) the use of public images for research purposes does not require consent.

The observation sample was a convenience sample (Anguera et al., 2001). Three thousand seven hundred and forty events have been collected corresponding to the observation of the 52 games that make up the world championship, specifically the group stage, round of 16, quarterfinals, semifinals, and the final. Of the total events, 780 have been discarded, corresponding to possessions that have ended in a draw. These matches are disputed in the direct elimination mode, which causes both teams to need offensive attack procedures to achieve a positive result.

### Observational Instrument

Three national football coaches and football research experts designed an *ad hoc* observation instrument, consisting of a combination of field format and category system, as proposed by Anguera et al. (2018). The proposed observation instrument can be consulted in Table 1.

**TABLE 1:** Dimensions that are part of the *ad hoc* instrument and derived category systems.

Criteria	Categories
Classification phase	Groups Round of 16 Quarterfinals Semifinals Final
Half time (match part)	First half
	Second half
Start form	Transition
	Set piece
Interaction context	AR: forward vs. delayed line
	AM: forward vs. middle line
	AA: forward vs. forward line
	MM: middle vs. middle line
	MR: middle vs. delayed line
	MA: middle vs. forward line
	RA: delayed vs. forward line
	RM: delayed vs. middle line
	PA: goalkeeper vs. forward line
Intention	Progress
	Keep
MD	Time the observed team keeps the ball in its defensive zone
MO	Time the observed team keeps the ball in its offensive zone
ZC	Zone in which the team maintained possession the most time
Time possession	Total possession duration
Passes	Number of total passes of the team possessing the ball
Move outcome	Goal
	Shot
	Send to area
	No success
Match status	Winning
	Drawing
	Losing
Final score	Win
	Draw
	Lose

### Data Notation

For criteria selection, different variables have been proposed empirically compared in previous works. The category definition for the *Interaction Context (COI)* variable can be consulted in Castellano (2000) and Castellano and Hernández-Mendo (2003). For the coding and registration of the *Possession Zone* and *Match Outcome* variables, the criteria proposed by Casal et al. (2017) were used. Regarding the *match status* variable, the proposal of Lago-Peñas and Dellal, 2010 has been used. For the registration and coding of the *Move outcome* variable, a modification of the proposal of Maneiro and Amatria (2018) and Maneiro et al. (2019a) has been made. Finally, the criteria used for the division of teams into two groups, successful and unsuccessful, has been the match outcome (win or lose), following the proposal of Lago et al. (2010) and Casal et al. (2017). In this way, all teams that won their matches were classified as successful and the teams that lost their matches as unsuccessful.

### Procedure

There were four observers selected for data collection, three of them being PhDs in Physical Activity and Sports Sciences, national football coaches and with more than 5 years of experience in the use and application of observational methodology. In addition, one of the authors is a methodologist and expert in observational methodology, with years of experience and relevant publications in the field (Losada and Manolov, 2015).

Prior to the coding process, the observers were trained during eight training sessions (Manolov and Losada, 2017.), applying the consensual agreement criterion among observers and they were provided with a specifically designed observation protocol. Data quality control was carried out using the IBM SPSS Statistics 25 program by means of an interobserver concordance analysis by Cohen’s (1960) Kappa coefficient for each of the criteria, the overall value being very good (0.83) according to the scales of Fleiss et al. (2003). For the rest of the analysis, the Compare Groups library of the R program version 3.4.2 was used.

### Statistic Analysis

To make the comparison between the “successful” and “not successful” groups in the women’s football teams, a first univariate analysis is started with the selected categorical variables, *Half.time, Start.form, COI, Intention, ZC* and *Move.outcome*, and the continuous *MD, MO, Time.possession* (in seconds) and *pass* (Tables 2, 4). To differentiate the groups “success” and “not success,” we used the variable *Final.score*, which has three categories w (victory), d (draw), and l (defeat). Of these, w (victory) was used to classify “successful” teams, and l (defeat) to classify “not successful” teams. It was checked if continuous variables followed a normal distribution, with the application of the Shapiro–Wilks test.

**TABLE 2:** Summary of results by groups of “match status.”

Variables	*N*	*p*-value	Method	Selection
1. Half.time	1230	< 0.001^∗∗^	Categorical	Final.score == “l”
2. Start.form	1230	0.933	Categorical	Final.score == “l”
3. COI	1230	.	Categorical	Final.score == “l”
4. Intention	1230	< 0.001^∗∗^	Categorical	Final.score == “l”
5. MD	1230	0.624	Continuous non-normal	Final.score == “l”
6. MO	1230	0.115	Continuous non-normal	Final.score == “l”
7. ZC	1230	0.433	Categorical	Final.score == “l”
8. Time.possession	1230	0.382	Continuous non-normal	Final.score == “l”
9. Pass	1230	0.421	Continuous non-normal	Final.score == “l”
10. Move.outcome	1230	.	Categorical	Final.score == “l”

Significant codes: 0 ‘**’ 0.05 ‘*’ 0.1 ‘ ’ 1.

Subsequently, a bivariate study was carried out, by groups, to find out the relationship between the variables described above, taking *Match.status* as a reference variable. For the set of categorical variables, chi-square tests or Fisher’s exact test were applied as necessary. The analysis was complemented by calculating its frequencies and corresponding percentage. In the case of the continuous variable, the median and values of Q1 and Q3 were calculated (Tables 3, 5). For continuous variables, time was collected in seconds.

**TABLE 3:** Bivariate descriptive summary with the variable “*Match.status*” as reference and the rest of the significant variables, in unsuccessful teams.

	dr	ls	Wn				
	*N* = 454	*N* = 740	*N* = 36	p.overall	p. dr. ls	p. dr. wn	p. ls vs. wn
Half.time				<0.001	<0.001	0.336	<0.001
ft	353 (77.8%)	230 (31.1%)	31 (86.1%)				
st	101 (22.2%)	510 (68.9%)	5 (13.9%)				
Start.form				0.933	1.000	1.000	1.000
sp	129 (28.4%)	217 (29.3%)	10 (27.8%)				
tr	325 (71.6%)	523 (70.7%)	26 (72.2%)				
COI				.	.	0.202	0.128
AA	15 (3.30%)	29 (3.92%)	0 (0.00%)				
AM	9 (1.98%)	10 (1.35%)	1 (2.78%)				
AR	5 (1.10%)	1 (0.14%)	0 (0.00%)				
MA	40 (8.81%)	74 (10.0%)	0 (0.00%)				
MM	276 (60.8%)	385 (52.0%)	26 (72.2%)				
MR	2 (0.44%)	0 (0.00%)	0 (0.00%)				
PA	62 (13.7%)	89 (12.0%)	3 (8.33%)				
RA	43 (9.47%)	147 (19.9%)	5 (13.9%)				
RM	2 (0.44%)	5 (0.68%)	1 (2.78%)				
Intention				<0.001	0.001	0.756	0.131
K	183 (40.3%)	219 (29.6%)	16 (44.4%)				
P	271 (59.7%)	521 (70.4%)	20 (55.6%)				
MD	8.00 [1.25;14.0]	8.00 [2.00;13.0]	7.50 [3.00;19.0]	0.624	0.612	0.612	0.612
MO	7.00 [2.00;12.8]	8.00 [3.00;13.0]	8.00 [3.75;14.0]	0.115	0.120	0.657	0.993
ZC				0.433	0.656	1.000	1.000
Offensive midfield	212 (46.7%)	374 (50.5%)	18 (50.0%)				
Defensive midfield	242 (53.3%)	366 (49.5%)	18 (50.0%)				
Time.possession	16.0 [11.0;22.0]	16.0 [12.0;23.0]	17.5 [14.0;23.2]	0.382	0.437	0.437	0.437
Pass	4.00 [3.00;6.00]	4.00 [3.00;6.00]	4.00 [3.00;6.25]	0.421	0.594	0.892	0.902
Move.outcome				.	0.192	1.000	1.000
EA	65 (14.3%)	149 (20.1%)	5 (13.9%)				
G	3 (0.66%)	7 (0.95%)	0 (0.00%)				
NE	325 (71.6%)	499 (67.4%)	26 (72.2%)				
S	61 (13.4%)	85 (11.5%)	5 (13.9%)				

Comparisons between pairs were studied applying *post hoc* tests, following a methodology described by Benjamini and Hochberg (1995).

**FIGURE 1:**
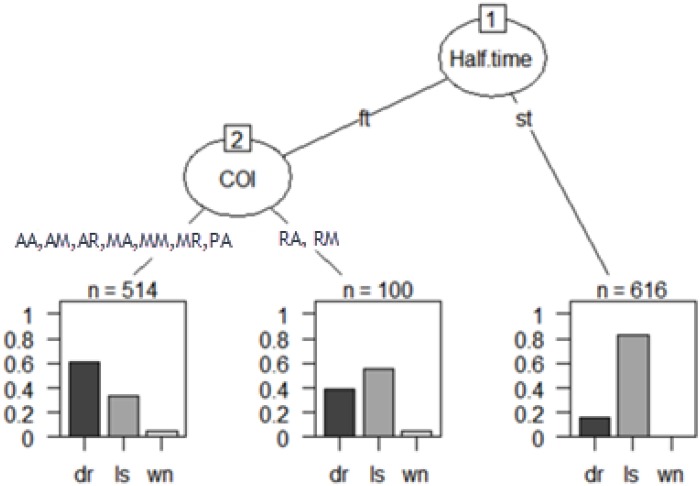
Decision tree: unsuccessful teams graph.

**FIGURE 2:**
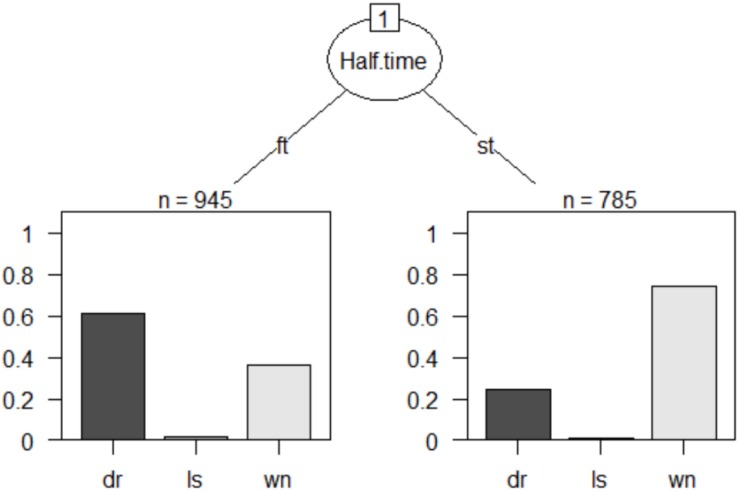
Decision tree: successful teams graph.

As a final analysis, a multivariable technique based on decision trees was incorporated (Figures 1, 2). It is a non-parametric approach, that is, without supposed distributions. It has an easy control of lost values, and strongly asymmetric data without the need to resort to data transformation. It is a robust analysis of outliers and, in addition, allows the analysis of sequential decisions based on the use of associated probabilities. For this, the chi-square automatic interaction detector (CHAID) was used as a growth method, which consists of a statistical and multidirectional tree algorithm that scans data quickly and efficiently, and creates segments and profiles compared to the desired result. In each step, CHAID chooses the predictor variable that presents the strongest interaction with the explained variable (Maneiro et al., 2019c). The categories of each predictor merge if they are not significantly different from the predictive variable.

## Results

The analysis begins with a study in non-successful teams, to select those variables that show differences comparing to the variable “*Match.status*” in unsuccessful teams. In this case, variables that show significance are: “*Half.time*,” with *p* < 0.001 and “*Intention*” with *p* < 0.001 (Table 2).

The variables “*MD*,” “*MO*,” “*Time.possession*” and “*Pass*,” showed a *p*-value <0.001 in the Shapiro–Wilks test, indicating that they do not follow a normal distribution.

Secondly, the Pearson’s chi-squared test was carried out to find out if there are significant differences in ball possession between successful (*n* = 1,730) and unsuccessful (*n* = 1,230) teams. In this case, results show that there are significant differences (*p* ≤ 0.000) between both groups considered.

In the bivariate study, for the “unsuccessful” teams, the criterion variable *Match.status* has been taken as a reference, given the relevance observed in other studies. As remarkable results we can see that the *Half.time* variable shows significant differences between the categories ft and st (<0.001). These differences are observed between the pairs “lose–draw” (0.001), and “lose–win” (0.001). In the *Start.form* and *COI* variable, no significant differences were observed. Significant differences exist in the *Intention* variable (<0.001), found in the pair “draw–lose” (0.001). In applied terms, Match status significantly influences half.time and tactical intent. In the case of *MD, MO, ZC, Time.possession*, *Pass* and *Move.outcome* variables, no significant differences were found for the levels of the *Match.status* category (Table 3).

Finally, a multivariate analysis was carried out using decision trees for unsuccessful and successful teams, where all study variables were included (Figure 1).

The result obtained for unsuccessful teams was collected in the theoretical tree:

[1] root

|  [2] Half.time in ft

|  |  [3] COI in AA, AM, AR, MA, MM, MR, PA: dr (*n* = 514, err = 38.9%)

|  |  [4] COI in RA, RM: ls (*n* = 100, err = 44.0%)

|  [5] Half.time in st: ls (*n* = 616, err = 17.2%)

Number of inner nodes: 2

Number of terminal nodes: 3

The first *root* node *Half.time* has two possibilities. The first is the category ft the algorithm includes a second node formed by the COI variable. This in turn is divided into two terminal nodes. In a sample of 514 possessions, and with a 38.9% error, with a draw marker (60% of the chances of having the ball), they have possession of the ball in the middle (MA, MM, and MR) and forward (AA, AM, and AR) field lines.

A second terminal node, with a sample of 100 possessions, and with an error of 44%, with an unfavorable match state, the possessions of the ball are recorded mainly in the delayed lines (RA and RM). During the second half, teams have more ball possessions with an unfavorable score (almost 80%).

The second category st derived from the first root node *Half.time* collects a single terminal node, where with a sample of 616 moves and an error of 17.2%. More than 80% of possessions, teams operations with the state of the game losing.

In the case of successful teams in women’s football, the analysis begins with a study to select those variables that show differences with respect to the variable “*Match.status*” in successful teams. In this case, the variables that show significance are: “*Half.time*,” with *p* < 0.001, “*MO*,” with *p* < 0.001, the “*ZC*” with a *p* = 0.003 and finally, the “*pass*” with a *p* = 0.017 (Table 4).

**TABLE 4:** Summary of results by groups of ‘*Match.status*.’

Variables	*N*	*p*-value	Method	Selection
1. Half.time	1730	< 0.001**	Categorical	Final.score == “w”
2. Start.form	1730	0.076	Categorical	Final.score == “w”
3. COI	1730	.	Categorical	Final.score == “w”
4. Intention	1730	0.830	Categorical	Final.score == “w”
5. MD	1730	0.179	Continuous non-normal	Final.score == “w”
6. MO	1730	< 0.001**	Continuous non-normal	Final.score == “w”
7. ZC	1730	0.003**	Categorical	Final.score == “w”
8. Time.possession	1730	0.102	Continuous non-normal	Final.score == “w”
9. Pass	1730	0.017**	Continuous non-normal	Final.score == “w”
10. Move.outcome	1730	.	Categorical	Final.score == “w”

Significant codes: 0 ‘**’ 0.05 ‘*’ 0.1 ‘ ’ 1.

In the “successful” female group, a Shapiro–Wilks was applied to the continuous variables “*MD*,” “*MO*,” “*Time.possession*” and “*Pass*” and a *p*-value <0.001 was obtained, confirming the lack of adjustment to normal distribution.

In the bivariate study, for the “successful” teams, as significant results in the variable *Half.time* significant differences (<0.001) exist, identified in the pairs draw–win (0.001) and lose–win (0.010). The MO variable presents significant differences (<0.001) and is manifested in the draw–win (<0.001), and lose–win (0.010) pairs. The ZC variable shows significant differences (0.003) and is found in the draw–win pair (0.007). The variable PASS presents a significant relationship (0.017), pertaining to the draw–win pair (0.041). In applied terms, it is possible to mention that the match status influences the ball possessions according to the match half (half.time), the zone of possession and the number of passes made.

Finally, the variables, “*Start.form*,” “*COI*,” “*MD*,” “*MO*,” “*ZC*,” “*Time.possession*,” “*Pass*,” and “*Move.outcome*” do not show significant differences between groups (Table 5).

**TABLE 5:** Bivariate descriptive summary with the variable “*Match.status*” as reference and the rest of the significant variables, in successful teams.

	dr	ls	Wn				
	*N* = 771	*N* = 28	*N* = 931	p.overall	p. dr. ls	p. dr. wn	p. ls vs. wn
Half.time				<0.001	0.269	<0.001	0.010
ft	581 (75.4%)	18 (64.3%)	346 (37.2%)				
st	190 (24.6%)	10 (35.7%)	585 (62.8%)				
Start.form				0.076	1.000	0.082	1.000
sp	229 (29.7%)	8 (28.6%)	231 (24.8%)				
tr	542 (70.3%)	20 (71.4%)	700 (75.2%)				
COI				.	0.124	.	0.031
AA	20 (2.59%)	1 (3.57%)	7 (0.75%)				
AM	11 (1.43%)	2 (7.14%)	17 (1.83%)				
AR	5 (0.65%)	1 (3.57%)	8 (0.86%)				
MA	101 (13.1%)	4 (14.3%)	93 (9.99%)				
MM	393 (51.0%)	12 (42.9%)	576 (61.9%)				
MR	1 (0.13%)	0 (0.00%)	1 (0.11%)				
PA	112 (15.7%)	6 (21.4%)	82 (8.81%)				
RA	121 (15.7%)	2 (7.14%)	138 (14.8%)				
RM	7 (0.91%)	0 (0.00%)	9 (0.97%)				
Intention				0.830	0.965	0.965	0.965
K	260 (33.7%)	11 (39.3%)	316 (33.9%)				
P	511 (66.3%)	17 (60.7%)	615 (66.1%)				
MD	9.00 [1.00;16.0]	9.00 [1.00;15.0]	7.00 [0.00;15.0]	0.179	0.838	0.193	0.838
MO	8.00 [4.00;13.0]	6.50 [3.00;10.0]	10.0 [6.00;15.0]	<0.001	0.134	<0.001	0.010
ZC				0.003	0.489	0.007	0.201
Offensive midfield	396 (51.4%)	12 (42.9%)	548 (58.9%)				
Defensive midfield	375 (48.6%)	16 (57.1%)	383 (41.1%)				
Time.possession	17.0 [12.0;25.0]	14.0 [11.0;17.2]	18.0 [12.0;26.0]	0.102	0.161	0.231	0.161
Pass	5.00 [3.00;7.00]	4.00 [2.75;6.00]	5.00 [3.00;7.00]	0.017	0.262	0.041	0.133
Move.outcome				.	0.260	0.655	0.260
EA	171 (22.2%)	5 (17.9%)	189 (20.3%)				
G	29 (3.76%)	4 (14.3%)	43 (4.62%)				
NE	466 (60.4%)	15 (53.6%)	565 (60.7%)				
T	105 (13.6%)	4 (14.3%)	134 (14.4%)				

In the case of successful teams, the theoretical tree was the following (Figure 2):

[1] root

|  [2] Half.time in ft: dr (*n* = 945, err = 38.5%)

|  [3] Half.time in st: wn (*n* = 785, err = 25.5%)

Number of inner nodes: 1

Number of terminal nodes: 2

The first *root* node *Half.time*, presents two possible nodes. The first is the category ft that, with 945 cases analyzed, and with an error of 38.5% is a terminal node and shows that during the first half they have more ball possessions than in the second half. In addition, 60% of possessions occur with the match status tying, and 40% winning. A second terminal node, configured by the st category, where 785 plays have been registered with an error of 25.5%, shows that successful teams make more than 75% of possessions with the winning match status, and only 25% drawing.

## Discussion

The present work was proposed with the objective of knowing the behavior of the match status variable and how it modulates the behavior of certain variables or behaviors in successful and unsuccessful teams in women’s football. For this, the FIFA Women’s World Cup 2015 has been analyzed, where the most representative and highest quality teams and players have met. The main novelties or contributions of this work could be summarized in two: the study and analysis of a sport still lacking robust scientific literature, such as women’s football; and a new multivariate technique in the world of football research, such as decision trees, has been put to the test (Maneiro et al., 2019c).

A first general approach, the available results allow us to think that significant differences exist between successful and unsuccessful teams regarding the “match status” variable in the three analysis performed. To facilitate the understanding and interpretation of this section, the results discussion will be presented respecting the order of appearance of the statistical techniques used.

At the univariate level, it is possible to mention that the match status modifies the ball possessions depending on the match half (half.time) in unsuccessful teams (Table 2). This result can be interesting, since knowing in what part of the field produces an imbalance in the scoreboard (a goal) can provide information to the teams when it comes to managing ball possession. This same result was also corroborated for the successful or winning teams (Table 4). The strength of the significance of this variable for both groups of teams (<0.001), suggests that ball possessions based on half.time are significantly influenced by match status. This result corroborates the previous study by Kirkendall et al. (2002) and Casal et al. (2017).

Although no significant results were found in terms of the beginning of the possession of the ball, there was a clear tendency to recover the ball by means of transition and not through set pieces. This result is in the line of previous works (Kirkendall et al., 2002), which state that 62% of ball possessions that end in goal are after a recovery in transition.

On the other hand, the results suggest that the tactical intention to progress or preserve is also significantly influenced by the match status in unsuccessful teams. This is something of value from a tactical point of view, because one of the most important decisions that teams must make when recovering the ball is whether to keep the same, by means of a gradual construction of the attack, or to progress directly toward the rival goal (Casal et al., 2017; Amatria et al., 2019a). As long as they opt for one option or another, in unsuccessful teams this will have a very important impact on the ball possession depending on whether it is winning, drawing, or losing.

As for successful teams, in addition to the variable “Half time,” there are three variables that have a statistically significant relationship with the match status variable: “MO,” “ZC,” and “Pass.” The match status can explain that successful teams have a greater number of ball possessions during the match, and they spend more time keeping the ball in their offensive zone than in the defensive zone, in general, and completing a greater number of passes in it. These results contradict the study of Paixão et al. (2015), who find that the successful teams in men’s football use longer pass sequences when they are losing; and the study by Casal et al. (2019), states that the match status variable does not modify the behavior of successful teams in high-level men’s football. On the other hand, it corroborates the study by Jones et al. (2004) on successful teams and ball possession.

In practical terms, the zone of possession is more important than possession by itself. In this case, that successful teams maintain possession in the opponent’s field presents a possible triple benefit: on the offensive level, it places the attacking team in areas close to the opponent’s goal, thus creating shooting possibilities on goal (Collet, 2013); and on a defensive level, Almeida et al. (2014) report that the best teams in football defend away from their own goal and close to a rival goal. On the other hand, it is important to remember that the teams that maintain possession in the offensive zone, increase the probability of winning by 1.72 times (Casal et al., 2017). It is likely that in this case successful teams in men’s football and women’s football share this same tactical behavior.

At the applied level, possession in the opponent’s field also presents two possible tactical interpretations that teams can take into account: first, a style of play based on possession in offensive zones allows better management of individual and collective efforts, and also allows to be close to the rival goal. On the other hand, the abuse and overuse of the possession resource in the opponent’s field can cause a reduction of the spaces between the different lines and between the different rival players, something potentially damaging to the interests of the attacking team, which can be found with multiple elements (rival players) in a very short space-time, which can limit the creativity of the most talented players. Talent needs space-time to express itself, for that reason constant ball possession in a opponent’s field could compress and reduce this aspect. Although this has hardly been studied in male football, spatio-temporal management is a fundamental aspect in sports of collective, complex and semi-chaotic nature such as football. Theoretical studies have begun to emphasize this aspect in collective sports (Araújo and Davids, 2016).

Finally, the number of passes in ball possession has also been erected as a variable that is modulated by the match status. The elaboration degree of possession has been significant in winning teams. The match status can explain that successful teams make a greater or smaller number of passes in their ball possessions depending on whether they are winning or losing. This result refutes the one found by Paixão et al. (2015), and corroborates that of Amatria et al. (2019b), which affirm that successful teams have higher elaboration levels (number of passes). This may be due to the creativity and talent of players, where high levels of elaboration create uncertainty in the rival’s defense, while feeding back their own potentialities. Another possible explanation, making a transfer of male to female football, is found in the work of Casal et al. (2017), which show that higher-level teams have longer possessions, and also the odds of winning a match is 44.25% with possession in the middle offensive zone.

Consequently, possible higher degree of elaboration. Other authors argue that it is due to the style of play implemented (Clemente, 2018). Grund (2012), in a study on male football in the Premier League, observed that the greater the number of passes, the greater the chances of scoring a goal, specifically 20% more.

Secondly, the bivariate analysis has confirmed that winning or successful teams perform significantly more possessions of the ball than unsuccessful or loser teams. These results seem to coincide with previous studies in men’s football (Casal et al., 2017; Amatria et al., 2019a), although the results should still be taken with caution since the results are still inconclusive (Jones et al., 2004; Lago et al., 2010).

The bivariate analysis has also allowed to extract information about the variables considered for ball possession and how they are modified by match status. In view of the results in Table 3, the losing or unsuccessful teams make very few ball possessions when the match status is winning (*n* = 36). In contrast, a large majority of possessions of these teams are made with the match status losing (*n* = 740). It is not possible to discuss these results with men’s football, because the unsuccessful-match status interaction has not been studied. Instead, one possible explanation may lie in the low quality of the losing teams regarding the winning teams, which make them fit goals easily and find themselves most of the time losing in the partial score.

Regarding the variables that explain match status, the *post hoc* test allowed us to measure the difference between pairs and thus compare where significant differences exist. As regards unsuccessful teams, there have been two significant variables: “half time” and “intention.” With respect to “half time,” significant differences appear between the draw vs. lose (<0.001) and the lose vs. win (<0.001) results. In the first half time, unsuccessful teams produce a greater number of possessions with the result of a draw (77.8%), while in the second half time greater possessions are produced while losing (68.9%). In other words, the match status may explain that teams that do not succeed accumulate their ball possessions in situations in which they are drawing, especially in the first half, and losing, especially in the second half. Although the absence of works on women’s football does not allow these findings to be discussed, similar results are found with respect to men’s football (Lago and Martín, 2007; Lago-Peñas, 2009). The explanations are multifactorial: from the style of play adopted by the team, which may vary depending on the match status; or possibly it is due to the tactical contingencies proper to the game, where the losing team needs the possession of the ball to be able to draw or win, since the score is against them. On the other hand, as regards the lose vs. win differences, the few ball possessions that unsuccessful teams make with the match status (match status) winning (*n* = 36), are produced in the first half. In contrast, in the second half, most of the possessions are made while losing. When a goal is scored and a favorable score is produced, they can not be consistent and maintain the result. A possible explanation for this behavior lies in the low quality of players, which causes the inability of unsuccessful teams to maintain a favorable result. Other possible causes lie in the physical and mental fatigue of the second half, or the style of play.

On the other hand, the match status also modulates the “intention” variable. A first interpretation of the results (Table 3) is that regardless of match status (winning, drawing, or losing), unsuccessful female teams opt for an intention to progress (Maneiro et al., 2019b), to the detriment of keeping or speculating with possession of the ball. This confirms a clear offensive and attacking will. These results disagree with those of Fernández-Navarro et al. (2018). Match status can also explain the intention of unsuccessful teams to progress more than to maintain possession of the ball. This is a congruent behavior, since with the scoreboard drawing or losing, unsuccessful teams must deploy offensive tactical progression to win the game. On the other hand, the *post hoc* test allowed us to compare the significant differences between the “draw vs. lose” pairs with respect to the “intention” variable. Losing teams are drawing in the first half, and losing in the second.

Regarding successful or winner teams, in view of the results of Table 5, a first reading is that ball possessions are never made with the match status losing (*n* = 28), while a large majority are made while winning (*n* = 931). These results disagree with those of Lago and Martín (2007) and Lago-Peñas (2009) in men’s football, although these studies do not differentiate between successful and unsuccessful at the end of the game. A possible explanation could be found in the great difference between the best and worst teams. The teams of more level and quality have greater facility to reach a favorable scoreboard than teams of lower level, and this makes most of the possessions coincide with a favorable score. A possible explanation could be found again in that best players compete in the best selections and are concentrated in specific countries.

Regarding the differences between pairs (Table 5), the *post hoc* test allowed to know that the “Half.time” is modulated by match status (*p* < 0.001), specifically in the draw vs. win (*p* < 0.001) and lose vs. win (*p* = 0.010) pairs. For the draw vs. win pair, successful teams perform a large part of the possessions with a draw score in the first half (75% possessions in a draw), while the main differences are in the second half, where almost 63% of ball possessions occur while winning, corroborating the work of Casal et al. (2019). These results could in turn explain the pair lose vs. win (*p* = 0.010), where successful teams always have better results in the second half. Some of the possible explanations are again the player quality of the best teams. In the first half, the talent and creativity of the best players and teams is matched by those of the worst teams thanks to some aspects such as physical effort, motivation, order, and match tactics. These abilities, applied with concentration and criteria, can allow to equate and compete against the best teams in the first half. On the other hand, the physical and mental fatigue of the second part does not allow to maintain these high physical and tactical standards, so the quality and talent of the best players and teams is decisive.

On the other hand, the numerical variable “MO” has been significant, specifically in the draw vs. win (*p* < 0.001) and lose vs. win (*p* = 0.010) pairs, which indicates that the match status may explain that successful teams maintain the ball more time in the mid-offensive zone (opponent’s field), and also in situations where they are drawing in comparison with winning in the first half, and winning in comparison with losing, in the second half. This may be due to the tactical and speculative management of possession, using the ball as a mechanism to avoid being attacked by the rival team. Also, during the first half they need ball possession because they are not yet winning, and during the second half, the longest possession time coincide with a favorable scoreboard. On the other hand, it should be taken into account that according to various studies, 74% of women’s football possessions begin in the offensive sector; and that ball possession in this offensive zone is a predictor of success (Kirkendall et al., 2002; Casal et al., 2019, respectively).

The categorical variable “ZC” has been significant in the draw vs. win (0.007) pair. Specifically, successful teams, with match status drawing, carry out a possession without prioritizing their own or opponent’s field (51.4% −48.6%). On the other hand, when successful teams pass from drawing to winning in match status, these differences are significantly increased in favor of the opponent’s field (58.9% −41.1%). One of the possible reasons may lie in the game style (Lago-Peñas, 2009) of teams. Another possible explanation, as mentioned above, could lie in the space-time needs (Pan et al., 2012) of players in their relationship with teammates and the ball.

Considering the principle of space-time as a vital necessity for the development of a successful possession of the ball, and that this principle can be reduced by the rival’s tactics, it is possible to affirm that possession of the ball with the intention of attacking may require its development in own field, and that possession of a ball with a defensive character may develop in a opponent’s field. In the first case, it aims to provoke the appearance of spaces behind the rival team. In the second case, the rival team is kept away from own goal. Therefore, the zone of ball possession does not indicate by itself the intentionality of the game, but rather it is the very purpose of possession that indicates what objectives it pursues. In short, ball possession in one’s own field or opponent’s field can serve as an instrument of deception and theatricality, inducing false leads to the rival team and camouflaging the true tactical intentionality.

Finally, the results are presented after the application of two decision trees (Figures 1, 2). With respect to the objectives of the study, it is possible to affirm that at quantitative level, the number of ball possessions does not vary between the first and the second part. On the other hand, there are significant differences between the lines of the team that perform the ball possession. More concretely, unsuccessful teams with a draw marker (60% of the possibilities that they would have the ball), have the possession of the ball in the middle (MA, MM, and MR) and forward (AA, AM, and AR) lines of the field. Whereas with an unfavorable match status, the ball possessions are registered mainly in the delayed lines (RA and RM). During the second half, teams have more ball possessions with an unfavorable score (almost 80%).

One possible explanation is that the matches start with a draw and it is logical that there are more possessions with the match status in a draw. In addition, possession of the ball is done by the offensive and middle lines, indicating the offensive and attacking will of the unsuccessful teams in the first half. In contrast, it is possible to observe how in the second part of the match, and with the unfavorable result, the interaction contexts are not significant. This could be due to the physical wear and tear of losing teams, which are not able to maintain possession in advanced or offensive lines. Despite the absence of multivariate work to discuss these findings, the work of Kirkendall (2007), highlighted the physical difference between players. From this work, we also highlight the tactical and technical difference between the best and worst teams of the FIFA Women’s World Cup 2015. These teams must improve their vision of the game, choose appropriate tactics for their interests and significantly increase their sports performance.

As regards successful teams (Figure 2), they present quantitatively greater number of ball possessions in the first half than in the second half. In addition, 60% of the possessions during the first half are made with the score in a draw, and 40% with the favorable score. Meanwhile, in the second half, teams make more than 75% of the ball possessions with the score winning. At the applied level, these results are antagonistic with respect to the losing teams. Again, it is plausible to think that winning teams have greater physical, technical and tactical skills than losing teams. In addition, they manage to maintain high performance standards during both parts of the match.

## Conclusion

The present study was intended to achieve a deeper knowledge, and with the adequate methodological and scientific support, on women’s football. For this, the variable “match status” has been taken as a reference variable, given its importance in scientific literature. It has sought to identify, characterize and differentiate different ball possessions between successful and unsuccessful teams of the past FIFA Women’s World Cup 2015. First, it has been shown that the number of ball possessions is a variable that presents differences statistically significant between successful and unsuccessful teams, and that this possession is strongly conditioned by the match status. The results have also shown significant differences between both groups of teams. Specifically, there are significant differences between winning and losing teams in terms of match status. Losing teams have more ball possessions in forward and middle lines with a draw during the first half, while in the second, a large percentage of possessions are made with an unfavorable match status. Instead, the successful teams have more ball possessions in the first part with a draw, while in the second it happens with a favorable match status.

Beyond these conclusions, women’s football is a phenomenon that is currently unstoppable and that is experiencing a great growth in recent years. This is allowing in turn to make women’s sport visible, to provide it with a professional structure and to propose tools and mobilize resources for its development. Therefore, it is essential that future research proposals revolve around this football, thus providing tools and scientific resources to coaches and players.

## Data Availability Statement

The datasets generated for this study are available on request to the corresponding author.

## Author Contributions

RM collected the data, reviewed the literature, and wrote the manuscript. JL performed the method and supervised the work critically. AA and CC supervised the work critically.

## Conflict of Interest

The authors declare that the research was conducted in the absence of any commercial or financial relationships that could be construed as a potential conflict of interest.
